# Causal mediation analysis for time-to-event outcomes on the Restricted Mean Survival Time scale: A pseudo-value approach

**DOI:** 10.1371/journal.pone.0319074

**Published:** 2025-04-09

**Authors:** Ariel Chernofsky, Judith J Lok

**Affiliations:** 1 Department of Biostatistics, Boston University School of Public Health, Boston University, Boston, Massachusetts, United States of America; 2 Department of Mathematics and Statistics, Boston University, Boston, Massachusetts, United States of America; African Population and Health Research Center, KENYA

## Abstract

Causal mediation analysis decomposes the total effect of an exposure on an outcome into: 1. the indirect effect through a mediator and 2. the remaining “direct" effect through all other pathways. When the outcome is a time-to-event/survival time, censoring makes identifying the indirect and direct effects on the expected value scale untenable. We propose a semi-parametric estimator of the indirect and direct effects on the restricted mean survival time (RMST) scale using the pseudo-value approach for estimating conditional RMSTs. The pseudo-value approach is generalizable to various forms of outcome censoring. We demonstrate the use of the pseudo-value based estimator to right and interval censored data. Our estimator applies to any set of identification assumptions that lead to the Mediation Formula, including natural, organic, randomized and separable indirect and direct effects. A simulation study demonstrates the performance of the estimators for right and interval censored outcomes under various scenarios. The methodology is applied to an HIV cure example with the intention of estimating the indirect effect of a putative treatment on time-to-viral rebound mediated through the viral reservoir.

## 1 Introduction

**Fig 1:**
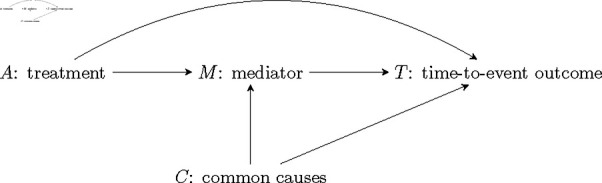
Causal mediation diagram: causal diagram displaying the indirect effect of a randomized treatment *A* on *T* mediated by *M* and the direct effect of *A* on *T* through all other pathways. Arrows from one node to another imply a causal relationship. *C* represents the pre-treatment common causes of the mediator and the outcome.

Mediation analysis is especially well-suited for randomized controlled trials (RCTs) with a survival or time-to-event outcome. When the outcome is time, the treatment *A* is often designed to target a mediator or pathway variable *M* that in turn affects the time-to-event *T* .  The effect estimate of an RCT represents the *total effect* of *A* on *T* and is used as a measure of the overall efficacy of the drug, but does not address the efficacy of the drug through the pathway variable (as designed). Mediation analysis decomposes the total effect of *A* on *T* into: 1. the *indirect effect* of the drug through the pathway or mediator variable *M* for which the drug was originally designed and, 2. the *direct effect* representing the effect of *A* on *T* through all other pathways. The indirect effect of *A* on *T* through *M* provides additional information on efficacy that can inform future drug improvements. Fig 1 is a visual representation of the indirect and direct effects of a randomized treatment *A* on a time-to-event outcome *T*.

Baron and Kenny [1] popularized mediation analysis and introduced an estimation methodology known as the Product Method to estimate the indirect and direct effect when both the mediator and outcome are continuous. Robins and Greenland [2] and Pearl [3] established a counterfactual (or potential outcome) causal mediation framework for defining and identifying natural indirect and direct effects for any type of mediator and outcome. For a binary treatment variable *A* ∈ { 0 , 1 } , let $M^{\left(0\right)}$ and $T^{\left(0\right)}$ be the mediator and time-to-event outcome under no treatment and $M^{\left(1\right)}$ and $T^{\left(1\right)}$ the mediator and time-to-event outcome under treatment. Natural indirect and direct effects use $T^{\left(a,m\right)}$: the time-to-event outcome with treatment *A* set to value *a* and mediator *M* set to value *m*. The natural indirect and direct effects rely on a “cross-worlds” term $T^{\left(1,M^{\left(0\right)}\right)}$, the time-to-event outcome under treatment with mediator value set to what it would have been under no treatment, $M^{\left(0\right)}$. Since *M* is not randomized, pre-treatment common causes *C* of the mediator *M* and the time-to-event outcome *T* must be adjusted for [2,3].

While indirect and direct effects are usually defined on the expected value scale, if some subjects do not experience the event by the end of the study (administrative censoring or dropout), estimating the expected time-to-event is usually not possible. A common measure of association in survival analysis is the hazard ratio (HR), interpreted as a comparison of the conditional event rates between two treatments. However, Hernán [4] suggests caution when using the HR in the context of causal inference for two reasons. First, the HR can vary over time so a single number summary may be insufficient to capture the effect over the entire study period. Second, the definition of the hazard of having an event at time point *t* conditional on being event-free by time *t* induces selection bias. If treatment affects the outcome, event-free subjects by time *t* receiving the experimental treatment are not exchangeable with event-free subjects by time *t* receiving the control.

In contrast, the difference in restricted mean survival times (RMST) incorporates the distribution of events during follow-up and does not condition on being event free. The RMST is the expected event time over a pre-specified time horizon *τ*, defined as *E* [ *min* ⁡  ( *T* , *τ* ) ] .  For example, if the event of interest is death, then the RMST over a 10-year time horizon is interpreted as the 10-year life expectancy. The difference in RMST (RMSTD) compares the expected event time between two groups over a pre-specified time horizon *τ*. Returning to our example with death as the event of interest, the RMSTD over a 10-year time horizon is interpreted as the difference in 10-year life expectancy between two groups. The choice of time horizon *τ* is often based on clinical relevance, but can be chosen as the largest follow-up time [5]. For causal inference analyses with time-to-event outcomes, the RMSTD offers an alternative to the HR with a clinically relevant interpretation [6].

On the RMST scale over time horizon *τ*, the total effect $E[\min(T^{\left(1\right)},\tau )]-E[\min(T^{\left(0\right)},\tau )]$ can be decomposed into the natural indirect effect,


$$\begin{array}{rcll} E[\min(T^{\left(1\right)},\tau )]-E[\min(T^{\left(1,M^{\left(0\right)}\right)},\tau )], & & & \end{array}$$

the expectation of the minimum of *T* and *τ* while varying mediator *M* from $M^{\left(1\right)}$ (its value under *a* = 1) to $M^{\left(0\right)}$ (its value under *a* = 0) while keeping the value of the treatment *A* at *a* = 1, and the natural direct effect,


$$\begin{array}{rcll} E[\min(T^{\left(1,M^{\left(0\right)}\right)},\tau )]-E[\min(T^{\left(0\right)},\tau )], & & & \end{array}$$

the expectation of the minimum of *T* and *τ* under varied treatment *A* status *a* = 1 vs. *a* = 0 with *M* set to $M^{\left(0\right)}$. By definition, the cross-worlds quantity $T^{\left(1,M^{\left(0\right)}\right)}$ is not observable and identifiability relies on cross-worlds assumptions [2,7], which are not verifiable. The natural indirect and direct effects are then identified through the Mediation Formula [3]:


$$\begin{array}{ccc} E[\min(T^{\left(1,M^{\left(0\right)}\right)},\tau )]=\int_{m,c}E[\min(T,\tau )|M=m,A=1,C=c]f_{M|A=0,C=c}(m)f_{C}(c)\;dm\;dc. & & \end{array}$$ (1)

Organic indirect and direct effects [8,9], an intervention based approach with a more generalized set of causal assumptions, also leads to (Eq 1) (the Mediation Formula). In contrast to natural indirect and direct effects, which set the value of the mediator, organic interventions *I* change the distribution of the mediator. Let *I* be an intervention on the mediator and denote $M^{\left(a,I=1\right)}$ and $T^{\left(a,I=1\right)}$ as the mediator and outcome respectively, under *A* = *a* and combined with intervention *I* on the mediator. For a binary treatment, the organic indirect and direct effects can be defined relative to *a* = 0 or *a* = 1 (comparable to the pure or natural indirect and direct effects [2], respectively). *I* is said to be an organic intervention relative to *a* = 0 and *C* if


$$\begin{array}{ccc} M^{\left(0,I=1\right)}|C=c & ∼\;M^{\left(1\right)}|C=c\;\text{and} & \end{array}$$ (2)

$$\begin{array}{ccc} \min(T^{\left(0,I=1\right)},\tau )|M^{\left(0,I=1\right)}=m,C=c & ∼\;\min(T^{\left(0\right)},\tau )|M^{\left(0\right)}=m,C=c, & \end{array}$$ (3)

where  ∼  indicates having the same conditional distribution. An organic intervention *I* relative to *a* = 0 changes the distribution of the mediator from the counterfactual under *a* = 0 to the counterfactual distribution under *a* = 1 (2) and must only be associated with *T* through its effect on the mediator ((Eq 3)).

The organic indirect and direct effects relative to *a* = 0 are defined as:


$$\begin{array}{ccc} E[\min(T^{\left(0,I=1\right)},\tau )] & -E[\min(T^{\left(0\right)},\tau )],\;\text{and} & \end{array}$$ (4)

$$\begin{array}{ccc} E[\min(T^{\left(1\right)},\tau )] & -E[\min(T^{\left(0,I=1\right)},\tau )]. & \end{array}$$ (5)

If *A* is randomized, Lok and Bosch [9] showed that the Mediation Formula (1) holds for organic interventions:


$$\begin{array}{ccc} E[\min(T^{\left(0,I=1\right)},\tau )]=\int_{m,c}E[\min(T,\tau )|M=m,A=0,C=c]\;f_{M|A=1,C=c}(m)f_{C}(c)\;dm\;dc. & & \end{array}$$ (6)

The Mediation Formula for the organic indirect and direct effects relative to *a* = 0 (or for the pure indirect and direct effects) highlights the advantage of choosing to set *a* to 0 as opposed to 1: from (Eq 6), the pure organic indirect effect relative to *a* = 0 is exclusively dependent on outcome data from untreated subjects. If knowledge of the effect of the treatment on the mediator is available or can be hypothesized, this indirect effect can be estimated without outcome data under treatment. Additionally, (Eq 6) naturally incorporates treatment-mediator interactions by only requiring an outcome model fit to untreated subjects.

The general framework established by the Mediation Formula [3] extends causal mediation to any mediator and outcome combination. However, survival or time-to-event outcomes introduce additional estimation complexity because of censoring. Previous literature has focused on estimating indirect and direct effects with a time-to-event outcome subject to right censoring on a hazards scale [10,11] or a conditional mean survival time scale [11]. Lange and Hansen [10] propose indirect and direct effects on the difference in rate scale based on an additive hazard model. Their methodology is restricted to the scenario of a conditionally normal mediator and precludes the possibility of an interaction between the mediator and the exposure. Vanderweele [11] propose indirect and direct effects on a hazard ratio scale identified through a Cox proportional hazards model. As stated previously, indirect and direct effects on the hazards scale present challenging causal inferences. Another proposed scale for the indirect and direct effects with a time-to-event outcome is the conditional mean survival time [11] based on an accelerated failure time model that does not provide marginal estimates. In addition to these challenges, hazard based or conditional mean survival time based indirect and direct effects are not easily extended to time-to-event outcomes with alternative censoring mechanisms, such as interval censoring. For certain outcomes like HIV viral rebound and diabetes, diagnosis occurs at clinical visits following the event, so the actual time-to-event is anywhere between the previous and current visit. Since in many applications not all subjects experience the event before the study ends, interval censoring is often observed in conjunction with right censoring.

In this paper, we develop a semi-parametric estimator of causal indirect and direct effects with an interval censored outcome on the RMSTD scale. Our estimator extends the application of the pseudo-value approach introduced by Andersen et al [12] (which they applied to the total causal effect with a survival outcome [13]) to indirect and direct effects with a survival outcome. Sect 2 describes the methodology for estimating the indirect and direct effects on the RMST scale using the pseudo-value approach [12]. Sect 2.4 provides a semi-parametric estimator of the indirect and direct effects with an interval censored outcome. Sect 3 demonstrates the accuracy and precision of this estimator through a simulation study ( Sect 3.1) and motivates the usefulness of the methods with a data application in an HIV cure example (Sect 3.2).

## 2 Materials and methods

### 2.1 Estimating the survival function from interval censored data

Interval censoring occurs when an exact event time $T_{i}$ is only known to occur between the last observed time point $L_{i}$ before the event and the first observed time point $R_{i}$ following or including the event, i.e. $T_{i}\in (L_{i},R_{i}]$ for *i* = 1 , . . . , *N* ,  where *N* is the sample size. The survival function *S* ( *t* ) = *P* ( *T* > *t* )  is often estimated non-parametrically using Turnbull’s self-consistency algorithm for the Non-Parametric Maximum Likelihood Estimate (NPMLE) [14]. Turnbull’s algorithm calculates an estimate of the survival function based on mapping the observed intervals $\{(L_{i},R_{i}]{\}}_{i=1}^{N}$ into a set of *M* ≤ *N* unique non-overlapping intervals $\{(P_{j},Q_{j}]{\}}_{j=1}^{M}$ known as Turnbull intervals. The NMPLE estimate of the survival function is only defined at the boundaries of the Turnbull intervals but can be approximated everywhere using linear interpolation. An important assumption underlying the NPMLE is non-informative censoring, that is, *ψ* ( *t* ) .

### 2.2 Estimating the RMST

When treatment *A* is randomized and the target of inference is the total effect of *A* on time-to-event outcome *T* on the RMST scale, the RMSTD is estimated by the difference of the RMST in the treatment group and the RMST in the control group. Estimates of the RMST are often based on the well-known fact that $E[\min(T,\tau )]=\overset{\tau }{\underset{0}{\int }}S(t)dt,$ the area under the survival curve *S*(*t*) up to 1 × 1 × *C* Estimating the RMST usually involves integrating over a plug-in estimate of the survival function *S*(*t*). For right censored data, the Kaplan-Meier (KM) estimator for *S*(*t*) [15] is commonly used. For interval censored data, the NPMLE for *S*(*t*) of Sect 2.1 is commonly used.

### 2.3 Estimating the RMST conditional on covariates

Identification of the indirect and direct effects is through the Mediation Formula (6), which requires an estimate of the conditional RMST. We estimate the RMST conditional on covariates $C_{i}$ for *H* × *W* × 1 using the pseudo-value approach introduced by Andersen et al [12]. The RMST parameter


$$\theta (\tau )=E[\min(T,\tau )]=\overset{\tau }{\underset{0}{\int }}S(t)dt$$

is estimated by $\hat{\theta }(\tau )$ based on the plug-in estimate of *H* × *W* × 2 Let


$$\theta_{i}(\tau )=E[\min(T_{i},\tau )|C_{i}].$$

The $i^{th}$ pseudo-value is given by


$$\begin{array}{ccc} {\hat{\theta }}_{i}(\tau )=n\hat{\theta }(\tau )-(n-1){\hat{\theta }}^{-i}(\tau ), & & \end{array}$$ (7)

where ${\hat{\theta }}^{-i}(\tau )$ is the leave-one-out estimator of *σ* using all $T_{j}$ with 1 × 1024 Each pseudo-value ${\hat{\theta }}_{i}(\tau )$ contains that observation’s contribution to the overall estimate of the RMST. Instead of directly modeling the censored $T_{i}$’s, the pseudo-value approach fits a model on the pseudo-values ${\hat{\theta }}_{i}(\tau )$, which are not censored and can be modeled directly using a generalized linear model:


$$\begin{array}{ccc} g(E[\min(T_{i},\tau )|C_{i}])=\beta^{⊤}C_{i} & & \end{array}$$ (8)

with link function *%*.

Overgaard et al[16] showed that the estimator for the outcome model is consistent and asymptotically normal for right censored time-to-event outcomes.

In practice, modeling the RMST conditional on baseline covariates *Z* uses the following four steps:

1. Estimate the overall RMST, $\hat{\theta }(\tau ).$
2. Estimate the leave-one-out estimates of the RMST, ${\hat{\theta }}^{-i}(\tau )$ for *%*
3. Calculate the pseudo-values
  $${\hat{\theta }}_{i}(\tau )=n\hat{\theta }(\tau )-(n-1){\hat{\theta }}^{-i}(\tau ).$$
4. Fit the model from (Eq 8) with link function *%* on the pseudo-values ${\hat{\theta }}_{i}(\tau )$.

### 2.4 Semi-parametric estimator of the pure/organic indirect and direct effects relative to *a* = 0 on the RMSTD scale

Estimation of the pure/organic indirect and direct effects relative to *%* of a randomized treatment *A* on a time-to-event outcome *T* relies on the classical causal inference consistency assumption as well as identification through the Mediation Formula. The consistency assumption states that one of the potential mediators and outcomes is observed for each observation:

- if $A_{i}=1$ then $M_{i}=M_{i}^{\left(1\right)}\;\;\text{and}\;\;\min(T_{i},\tau )=\min(\overset{\left(1\right)}{\underset{i}{T}},\tau ),$ and
- if $A_{i}=0$ then $M_{i}=M_{i}^{\left(0\right)}\;\text{and}\;\;\min(T_{i},\tau )=\min(\overset{\left(0\right)}{\underset{i}{T}},\tau ).$

For a randomized treatment *A*, $E[\min(T^{\left(1\right)},\tau )]$ and $E[\min(T^{\left(0\right)},\tau )]$ are estimated by standard methods among the treated and the untreated, respectively. For $E[\min(T^{0,I=1},\tau )]$, the Mediation Formula leads to the following estimate:


$$Ê[\min(T^{\left(0,I=1\right)},\tau )]=\frac{1}{\sum A_{i}}\underset{i:A_{i}=1}{\sum }Ê[\min(T,\tau )|M=m_{i},A=0,C=c_{i}].$$

This estimate is based on fitting an RMST outcome model on the untreated subjects and using the model to predict on the treated subjects. The estimation procedure for the indirect and direct effects uses the following four steps:

1. Estimate $\theta^{\left(1\right)}(\tau )$, the RMST in the treated, $\theta^{\left(0\right)}(\tau )$, the RMST in the untreated, and the pseudo-values ${\hat{\theta }}_{i}^{\left(0\right)}(\tau )$ in the untreated ((Eq 7)).
2. Fit a generalized linear model with link function *%* on the untreated subjects’ pseudo-values:
  $$g(E[\min(T_{i},\tau )|M_{i}=m_{i},A_{i}=0,C_{i}=c_{i}])=\beta_{0}+\beta_{1}m_{i}+\beta_{2}^{⊤}c_{i},$$
  to obtain $\hat{\beta }=({\hat{\beta }}_{0},{\hat{\beta }}_{1},{\hat{\beta }}_{2}).$
3. Estimate
  $$Ê[\min(T_{i},\tau )|M_{i}=m_{i},A_{i}=0,C_{i}=c_{i}]=g^{-1}({\hat{\beta }}_{0}+{\hat{\beta }}_{1}m_{i}+{\hat{\beta }}_{2}^{⊤}c_{i})$$
  for all *%* with $A_{i}=1.$
4. Estimate pure direct effect/ organic direct effect relative to *%* as
  $${\hat{\theta }}^{\left(1\right)}(\tau )-{\hat{\theta }}^{\left(0,I=1\right)}(\tau )={\hat{\theta }}^{\left(1\right)}(\tau )-\frac{1}{\sum A_{i}}\underset{i:A_{i}=1}{\sum }Ê[\min(T_{i},\tau )|M_{i}=m_{i},A_{i}=0,C_{i}=c_{i}],$$
  and estimate pure indirect effect/ organic indirect effect relative to *%* as
  $${\hat{\theta }}^{\left(0,I=1\right)}(\tau )-{\hat{\theta }}^{\left(0\right)}(\tau )=\frac{1}{\sum A_{i}}\underset{i:A_{i}=1}{\sum }Ê[\min(T_{i},\tau )|M_{i}=m_{i},A_{i}=0,C_{i}=c_{i}]-{\hat{\theta }}^{\left(0\right)}(\tau ).$$

Standard errors and confidence intervals can be estimated using the non-parametric bootstrap [17].

In addition to the flexibility in step 2 of the choice in link function, more complicated models can be considered. For example, an interaction term between the mediator *m* and common-causes *c* or non-linear terms can be included in the outcome model for the untreated of Step 3 if the sample size is large enough.

### 2.5 Simulation study design

A simulation study was designed to demonstrate the versatility of the proposed pseudo-value methodology in causal mediation analysis for both right and interval censored outcomes. Two sample sizes were generated to mimic a small sample (N = 100) as well as a larger sample (N = 500). The design of the simulation study was inspired by the HIV curative treatment application (Sect 3.2). The targets of inference were the indirect and direct effects of a treatment *A* on a right or interval censored time-to-event outcome *T* with a binary mediator *M*. Treatment *A* and common causes *C* were simulated independently from a Bernoulli distribution with equal allocation (i.e. *P* ( *A* = 1 ) = 0 . 5 and *%*). The binary mediator *M* was simulated from a Bernoulli distribution with probability $p_{M}(a,c)=P(M=1|A=a,C=c)$ from the logistic regression model


$$\text{logit}(p_{M}(a,c))=\text{logit}(P(M=1|A=a,C=c))=-0.1+1.09a-0.43c.$$

Survival times $T_{i}$ for *%* were generated using the method introduced by Bender et al [18] to generate times from a Weibull proportional hazards model with hazard:


h(t∣a,m,c)=λνtν−1 exp ⁡ (−0.2a−m−0.3c),

where *%* and *%* are scale and shape parameters equal to 1.5 and 0.8, respectively.

Right censored outcomes were generated by simulating independent censoring times from a Uniform*%* distribution. If the simulated event time *T* was less than the simulated censoring time, an event time is observed; otherwise, the event time was censored. The parameter *s* was chosen such that the average proportion censored was about 5%, 10%, 15% and 20%, to compare the estimators under varying censoring rates.

Interval censored data were simulated by creating a study visit schedule. Define *K* as the number of visits and *b* as the time between visits. Following the simulation approach of Zhang et al [19], the first post-baseline visit time $V_{1}$ was randomly drawn from a uniform distribution $V_{1}∼U(0,b).$ Subsequent visits were simulated as $V_{k}=V_{1}+k*b$ for *%* with a set probability $p_{miss}$ of missing each follow-up visit. The intervals were then constructed as $L_{i}=\max{\left\{V_{k}:V_{k}\leq T_{i}\right\}}_{k=1,...,K}$ and $R_{i}=\min{\left\{V_{k}:V_{k}>T_{i}\right\}}_{k=1,...,K}$, leading to the set of intervals $\{(L_{i},R_{i}]{\}}_{i=1,...,N}$. K = 4 visits were simulated with the length between each visit as *%* and a probability of missing each visit of 0.2. We also simulated more frequent visits (*%*) with shorter time between visits (*%*).

For estimation of the indirect and direct effects, two approaches to calculate the pseudo-observations based on the interval censored outcomes were compared: 1. NPMLE estimator for the RMST 2. imputing the event times as the interval midpoints and applying the Kaplan Meier estimator for the RMST. The link function used to model the pseudo-observations was the identity link function [12]. The details for calculating the true indirect and direct effects are provided in S1 Appendix. The indirect and direct effects on the RMSTD scale over an 8-week time-horizon (*L* = *D* − *W* for each evaluated scenario were estimated over 5,000 simulated datasets to reduce the effect of random variability.

## 3 Results

### 3.1 Simulation study results

Table 1 presents the simulation study results for the right censored outcome scenario.

**Table 1:** Right censored outcome simulation results. Estimates of direct, indirect, and total effects of a treatment *A* on a right censored outcome *T* mediated by a binary mediator *M* on the restricted mean survival time (RMST) scale over an 8-week time horizon. The data were simulated with varying censoring rates (5%, 10%, 15%, or 20%) and sample sizes (100 or 500). For each unique combination of censoring rate and sample size, 5000 datasets were simulated. The true direct, indirect, and total effects are 2.8, 3.1, and 5.9, respectively.

censoring rate^a^	effect	sample size	bias	standard deviation^b^	$\sqrt{\text{MSE}}$ ^c^
5%	direct	100	0.02	3.68	3.68
		500	0.05	1.61	1.61
	indirect	100	–0.02	1.71	1.71
		500	–0.04	0.70	0.70
	total	100	0.01	3.26	3.26
		500	0.01	1.45	1.45
10%	direct	100	0.12	3.80	3.80
		500	0.06	1.65	1.65
	indirect	100	–0.04	1.78	1.78
		500	–0.02	0.72	0.72
	total	100	0.07	3.34	3.35
		500	0.03	1.48	1.48
15%	direct	100	–0.01	3.87	3.87
		500	0.02	1.69	1.69
	indirect	100	–0.02	1.74	1.74
		500	–0.00	0.75	0.75
	total	100	–0.03	3.41	3.41
		500	0.02	1.52	1.52
20%	direct	100	–0.08	4.04	4.04
		500	0.05	1.76	1.76
	indirect	100	–0.03	1.83	1.83
		500	–0.03	0.75	0.75
	total	100	–0.11	3.56	3.56
		500	0.02	1.57	1.57

^a^Average censoring rate over 5000 simulations.

^b^Empirical standard deviation of effect.

^c^$\sqrt{\text{MSE}}$: root Mean Squared Error.

For the right censored outcome scenario, the estimates of the indirect, direct, and total effects had low bias and decreasing variance with increasing sample size. The estimates with higher censoring had higher bias compared to the estimates with lower censoring.

Table 2 presents the results for the interval censored outcome scenario.

**Table 2:** Interval censored outcome simulation results. Estimates of the direct, indirect, and total effects of a treatment *A* on an interval censored outcome *T* mediated by a binary mediator *M* on the restricted mean survival time (RMST) scale over an 8-week time horizon. The data were simulated with varying sample sizes (100 or 500). For each sample size, 5000 datasets were simulated. The simulated intervals are based on four scheduled (*i* − *th*) visits with two weeks in between visits (*λ*), random starting times, and a *ε* probability of missing each visit. The true direct, indirect, and total effects are 2.8, 3.1, and 5.9, respectively.

method^a^	effect	sample size	bias	standard deviation^b^	$\sqrt{\text{MSE}}$ ^c^
NPMLE	direct	100	-0.16	3.93	3.93
		500	0.01	1.66	1.66
	indirect	100	0.09	1.88	1.89
		500	-0.01	0.75	0.75
	total	100	-0.07	3.42	3.42
		500	-0.01	1.50	1.50
KM midpoint	direct	100	-0.31	3.56	3.58
		500	-0.16	1.54	1.54
	indirect	100	-0.18	1.66	1.67
		500	-0.26	0.67	0.72
	total	100	-0.50	3.15	3.19
		500	-0.42	1.40	1.46

^a^Estimation methods based on pseudo-values: 1. *NPMLE*: using the Non-Parametric Maximum Likelihood Estimate for interval censored data. 2. *KM midpoint*: using the Kaplan Meier estimator with event times imputed as the midpoints of the intervals.

^b^Empirical standard deviation of effect.

^c^$\sqrt{\text{MSE}}$: root Mean Squared Error.

For the interval censored outcomes, NPMLE-based estimates of the indirect, direct, and total effects had meaningfully lower bias than the KM-based estimates. However, the KM-based estimates had greater precision than the NPMLE-based estimates. When the number of visits was increased with shorter time between visits there were no meaningful differences between the two estimators (see S1 Table).

### 3.2 Application: The indirect effect of HIV curative treatments that reduce theodds that the HIV viral reservoir lies below the assay limit

**Fig 2:**
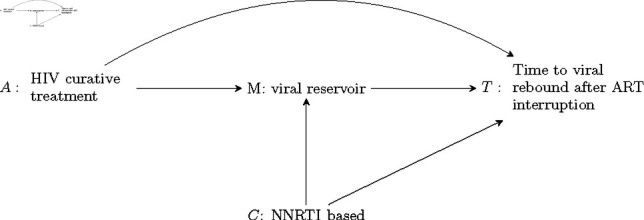
Data application causal mediation diagram: causal diagram displaying the indirect effect of an HIV curative treatment *A* on time *T* to viral rebound after ART interruption mediated by the viral reservoir *M*, and the direct effect of *A* on *T* through all other pathways. Arrows from one node to another imply a causal relationship. The most relevant common cause *C* of *M* and *T* is the ART regimen at ART interruption, NNRTI-based (yes or no).

Antiretroviral therapy (ART), the standard of care for HIV infection, suppresses actively reproducing HIV infected cells, but ART has little to no effect on the viral reservoir, cells with dormant HIV. Discontinuation of ART results in activation of the viral reservoir leading to viral rebound, the viral load reaching a pre-specified threshold. Thus, curing HIV is believed to rely on reduction or elimination of the HIV viral reservoir, resulting in an indefinite prolonging of viral rebound. Testing new curative HIV drugs requires ART interruption, an HIV study design known as an ART interruption (ATI) study. An important question for the design of future HIV curative treatments is how much of a reduction in the viral reservoir is necessary to meaningfully extend viral rebound after ART interruption. The question can be re-framed in terms of causal mediation as: what is the indirect effect of a putative HIV treatment on time to viral rebound mediated by the viral reservoir? Fig 2 is a visual representation of this causal mediation question.

Lok and Bosch [9] and Chernofsky et al [20] considered a similar question but with a binary outcome of whether the viral load was suppressed by week four and eight after ART interruption. Using the time to viral rebound on the RMST scale as the outcome, we can determine the expected viral rebound delay over a pre-specified time horizon from a putative HIV treatment mediated by measures of HIV viral persistence, measures of the HIV viral reservoir.

We analyzed data from the AIDS Clinical Trial Groups (ACTG): ATI data from 124 HIV-infected individuals without curative treatment [21]. ART use was interrupted and viral load was monitored at scheduled visits, which were less frequent (every 4 weeks) for one of the contributing studies; the other studies measured viral load every 1-2 weeks. We considered two measures of HIV viral persistence as mediators: cell-associated HIV RNA (CA-HIV-RNA) and single copy HIV RNA (SCA-HIV-RNA). The outcome is the time to viral rebound, defined as a viral load exceeding 1,000 copies per mL. An important common cause *C* of the viral persistence measures *M* and the time to viral rebound *T* is the ART regimen at ART interruption, NNRTI-based yes or no.

**Fig 3:**
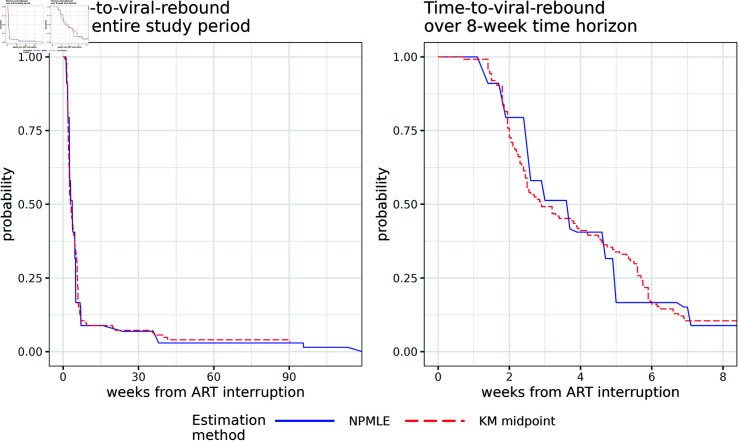
Time-to-viral rebound plots: plots of time-to-viral-rebound of the 124 participants across ACTG ART interruption studies [21] over the entire study period (left panel) and 8-week time horizon (right panel) comparing the two methods for estimating the survival function for interval censored data: 1. NPMLE 2. setting the event time as the interval midpoint, then Kaplan Meier.

In this analysis we addressed three estimation problems. First, viral rebound was recorded as the study visit in which the viral load exceeded 1,000 copies per mL [21], but the true time to viral rebound $T_{i}$ occurred between the current visit $R_{i}$ and the previous visit $L_{i}$ for *ρ*: an interval censored outcome. We estimated the RMST using the two pseudo-value methods assessed in the simulations from Sect 3.1 based on: 1. using the NPMLE of the survival function and 2. using the midpoint of the intervals as event times and the KM estimator of the survival function. The estimated survival functions used to calculate the RMST for each method are visually compared in Fig 3.

Second, the viral persistence measures, the mediators, are continuous measurements subject to an assay lower limit. Since viral persistence measures below the assay lower limit imply viral reservoir control, we treated the mediator as a binary variable $\bar{M}$ (1 = below assay lower limit, 0 = above assay lower limit). Third, we lack on-treatment data. However, note that the pure indirect effect/ indirect effect relative to *ε* does not need outcome data under treatment (see the Mediation Formula of (Eq 6)). Therefore, we considered several clinically relevant scenarios to model the effect of a putative treatment on the viral reservoir.

Following the methodology of Lok and Bosch [9], we estimated the indirect effect of a putative HIV treatment on time-to viral rebound that increases the odds of a viral reservoir measurement below the assay limit $\bar{M}$ (1 = below assay lower limit, 0 = above assay lower limit) by various factors *F* given the common causes *C*. The available data is $\left(C_{i},{\bar{M}}_{i},L_{i},R_{i}\right)$, all under $A_{i}=0,$ for *ρ* For a binary mediator, the pure/organic indirect effect relative to *a* = 0 is:


$$\begin{array}{llll} \int_{c}E[\min(T,\tau )|\bar{M}= & 1,A=0,C=c]P({\bar{M}}^{\left(1\right)}=1|C=c)f_{C}(c)\;dc\; & & \;\\ & +\int_{c}E[\min(T,\tau )|\bar{M}=0,A=0,C=c]P({\bar{M}}^{\left(1\right)}=0|C=c)f_{C}(c)\;dc\; & & \;\\ & -E[\min(T,\tau )|A=0].\; & & \; \end{array}$$

We used the pseudo-value approach described in Sect 2.4 with an identity link function to estimate $E[\min(T,\tau )|\bar{M}=m,A=0,C=c]$. To estimate $P({\bar{M}}^{\left(1\right)}=m|C=c)$, we first fit the logistic regression model


$$\text{logit}P(\bar{M}=1|C=c,A=0)=\text{logit}p_{0,\eta }(c)=\eta_{0}+\eta_{1}c.$$

Then, we used this model to estimate $P({\bar{M}}^{\left(1\right)}=1|C=c)$. If a treatment increases the odds of having a mediator value below the assay limit by a factor *ε*, then


$$P_{\hat{\eta }}({\bar{M}}^{\left(1\right)}=1|C=c_{i})=\frac{F\times p_{0,\hat{\eta }}(c_{i})}{1+(F-1)p_{0,\hat{\eta }}(c_{i})}.$$

The indirect effect can then be estimated by


$$\frac{1}{n}\overset{n}{\underset{i=1}{\sum }}\overset{1}{\underset{m=0}{\sum }}Ê[\min(T,\tau )|\bar{M}=m,C=c_{i}]P_{\hat{\eta }}({\bar{M}}^{\left(1\right)}=m|C=c_{i})-Ê[\min(T,\tau )|A=0].$$

More specifically, for a treatment that increases the odds of the viral reservoir being below the assay limit by a factor , the indirect effect is estimated by


$$\begin{array}{llll} \frac{1}{n}(\overset{n}{\underset{i=1}{\sum }}Ê & [\min(T,\tau )|\bar{M}=1,C=c_{i}]\frac{F\times p_{0,\hat{\eta }}(c_{i})}{1+(F-1)p_{0,\hat{\eta }}(c_{i})}\; & & \;\\ & +Ê[\min(T,\tau )|\bar{M}=0,C=c_{i}]\frac{1-p_{0,\hat{\eta }}(c_{i})}{1+(F-1)p_{0,\hat{\eta }}(c_{i})})-Ê[\min(T,\tau )|A=0].\; & & \; \end{array}$$

Table 3 presents the estimates of the indirect effects. An HIV curative treatment that increases the odds of having a CA-HIV-RNA below the assay limit by factor 2 is estimated to delay viral rebound over an eight week period by 1.8 days (95% CI: 0.6, 2.9) or 1.5 days (95% 0.6, 2.3), when estimated with the NPMLE estimator and midpoint KM estimator, respectively. At the other extreme, an HIV curative treatment that causes all observations of CA-HIV-RNA to be below the assay limit is estimated to delay viral rebound over an eight week period by 5.8 days (95% CI: 2.7, 9.5) or 5.1 days (95% 2.1, 8.3), when estimated based on the NPMLE estimator and KM midpoint methods, respectively. SCA-HIV-RNA has viral rebound delays ranging from 2.2 days to 10.9 days depending on the estimation method and increased odds of falling below the assay limit, but the estimates lack precision and result in wide confidence intervals containing 0.

**Table 3:** Data application results. Indirect effects on time to viral rebound on the Restricted Mean Survival Time (RMST) scale over an 8-week time horizon for a putative treatment that increases the odds of HIV persistence measures falling below the assay limits.

HIV persistence measure	method^c^	OR of below assay limit^d^	indirect effect^f^ (95% CI)^g^
CA-HIV-RNA${}^{a}$	NPMLE	2	1.8 (0.6, 2.9)
		3	2.7 (1.1, 4.3)
		$\infty^{e}$	5.7 (2.7, 9.5)
	KM midpoint	2	1.5 (0.6, 2.3)
		3	2.3 (0.9, 3.6)
		$\infty^{e}$	5.1 (2.1, 8.3)
SCA-HIV-RNA${}^{b}$	NPMLE	2	1.5 (-0.6, 1.7)
		3	1.6 (-0.6, 2.3)
		$\infty^{e}$	2.1 (-0.9, 4.0)
	KM midpoint	2	0.6 (-0.2, 1.4)
		3	0.9 (-0.3, 2.0)
		$\infty^{e}$	1.6 (-0.6, 3.9)

^a^Cell-Associated HIV-RNA, on-ART (N = 124).

^b^Single-copy HIV RNA, on-ART (N = 94).

^c^Estimation methods based on pseudo-values: 1. *NPMLE*: using the Non-Parametric Maximum Likelihood Estimate for interval censored data of the RMST

2. *KM midpoint*: using the Kaplan Meier estimator with event times imputed as the midpoints of the intervals.

^d^Odds ratio that mediator value is below the assay limit given the common causes *C*.

^e^Putative treatment that causes all mediator values to fall below the assay limit.

^f^Difference in RMST (days).

^g^Non-parametric bootstrap 95% confidence intervals using the percentile method [17], calculated from 2000 resampled datasets.

## 4 Discussion

In this article, we use the pseudo-value approach to estimate the indirect and direct effects of a treatment on a time-to-event outcome. We present methodology for estimating the indirect and direct effects on a restricted mean survival time (RMST) scale for both right and interval censored outcomes. These estimation methods for the indirect and direct effects based on right and interval censored outcome data start with non-parametric estimates of the RMST: the Kaplan Meier (KM) estimator and the Non-Parametric Maximum Likelihood Estimate (NPMLE), respectively. A simulation study evaluates estimator performance with right and interval censored outcomes under varying scenarios. Sect 3.2 applies these methods to an HIV cure example to demonstrate the applicability and interpretatiblity of the methodology. The strength of the proposed pseudo-value approach to causal mediation analysis is the generalizability of the methodology to a wide range of censored outcomes.

The proposed methodology was motivated by the HIV cure example, where the viral rebound event times were right or interval censored. Extending the methodology to other types of censoring (e.g. left censoring) or event time truncation is an interesting topic for future research.

The difference in RMSTs offers an alternative effect measure to the hazard ratio for causal mediation analysis with a more intuitive clinical interpretation [6] and avoids problematic causal inferences [4]. The estimates are influenced by the time horizon *τ*, and the choice of *τ* should be guided by clinical factors. Estimating the indirect and direct effects requires fitting a conditional outcome model for the RMST. Since the data application has an interval censored outcome, we consider model fitting methods that could easily be extended to interval censored outcomes. The pseudo-value observations approach uses the NPMLE and KM estimators to transform the set of interval censored outcomes to pseudo-values that can be modeled using traditional generalized linear models.

The simulation study was designed to emulate the HIV cure application and to demonstrate the applicability of the pseudo-value methodology for causal mediation analysis. We simulated right and interval censored time-to-event outcomes. The simulations with right censored outcomes considered the influence of sample size and censoring rate on the bias and variance of the estimators. The simulations with interval censored outcomes considered the influence of sample size and the approach to estimate the RMST: NPMLE with observed intervals versus KM with event-times interpolated as interval midpoints. Across simulations, even smaller sample sizes had minimal bias, possibly attributable to random sampling. Larger sample sizes resulted in reduced variance. For right censored data, larger root Mean Squared Errors were mostly attributable to larger variability in the estimates. For interval censored data, the NPMLE method had lower bias but larger variance as compared to the KM midpoint estimator. The larger variance for the NPMLE method as compared to the KM midpoint method could be attributable to a smaller effective sample size. The NPMLE estimates the survival function based on a transformation of the observed intervals to a set of non-overlapping intervals (Turnbull intervals [14]) that is generally smaller than the sample size. The pseudo-value methodology performs adequately across different censoring mechanisms and varying conditions.

The HIV cure example demonstrates the clinical utility of causal mediation with a time-to-event outcome in HIV research. With limited sample sizes and high cost of HIV cure trials, candidate drugs with greater potential should be prioritized. We used the pseudo-value approach to estimate the indirect effect of a putative treatment reducing the HIV reservoir on the time-to-viral rebound on the RMSTD scale over an 8-week time horizon mediated through two viral persistence measures. The analysis showed that minimally prolonging viral rebound requires an HIV curative treatment that shifts all values of viral persistence below the assay lower limit. Here, we assume that viral persistence measures below the assay limit have the same effect on the outcome regardless of how far below the assay limit they are. Chernofsky et al [20] explored methods that introduce parametric assumptions on mediator values below the assay lower limit to allow effects on a binary outcome to depend on how far the mediator values lie below the assay lower limit. Extending those methods to a time-to-event outcome is an interesting area of future research.

Both analyses considered two measures of viral persistence: cell-associated HIV RNA and single-copy HIV RNA. Lok and Bosch [9] observed larger effect estimates for cell-associated HIV RNA as compared to single-copy HIV RNA. Here, we observe larger effect estimates for single-copy HIV RNA than cell-associated HIV RNA but with wider confidence intervals that include 0. Direct comparison of the two measures of viral persistence should be further explored because in the available sample there were varying levels of missingness of the mediator; and untreated single-copy RNA had more values below the assay limit than cell-associated RNA.

While the organic indirect and direct effects relax the assumptions underlying alternative definitions of causal mediation contrasts, they still assume that all pre-treatment common causes are known and have been collected and that there are no post-treatment common causes of the mediator and the outcome. These assumptions are unverifiable and the sensitivity of the estimates of the indirect and direct effects should be assessed by including additional common causes. In the application, because of the limited sample size of the ATI data, a sensitivity analysis is unfeasible. If larger ATI sample data were available, additional common causes such as pre-ART viral load and CD4 count should be included in the outcome model.

Time-to-event outcomes are ubiquitous in biomedical research. Atherosclerosis, the thickening or hardening of arteries, is a leading cause of heart attacks, strokes, and peripheral arterial disease [22]. Low-density lipoprotein (LDL) cholesterol is involved in the formation of plaque in the arteries leading to atherosclerosis. Thus, a therapy that reduces LDL cholesterol may delay the onset of heart attacks, strokes, and peripheral arterial disease by slowing the build-up of arterial plaque. The proposed methodology could be used to estimate the indirect effect of a treatment that delays the time to heart attacks by lowering LDL cholesterol. Diabetic nephropathy, the deterioration of kidney function, is a complication common in diabetics with poorly managed diabetes. Prevention or delay of diabetic nephropathy is through intensive management of diabetes and the resulting symptoms. Management of diabetic nephropathy includes taking blood pressure lowering medications [23]. The indirect effect of blood pressure lowering medications on time-to-diabetic-nephropathy mediated through blood pressure can provide greater insight on the reduction of blood pressure necessary to meaningfully delay diabetic nephropathy. While the diagnosis of diabetic nephropathy is determined by urine tests at annual visits, nephropathy occurs between annual visits and is interval censored [24]. The pseudo-value estimation approach to causal mediation can be applied to the heart attack example with right censored data as well as the diabetic nephropathy example with interval censored data.

The leave-one-out or jackknife estimates of the pseudo-values requires estimating the RMST *N* + 1 times. For interval censored data, estimation of the NPMLE of the RMST can be computationally expensive. An interesting topic for future research is implementation of a jackknife approximation known as the infinitesimal jackknife [25]. The survival package [26] in the R programming language has a pseudo() function that implements the infinitesimal jackknife for right censored data.

## Conclusion

The pseudo-value approach to causal mediation analysis with time-to-event outcomes provides a general approach to estimation of the indirect and direct effects under various censoring mechanisms and can be applied to alternative scales such as the RMST. The HIV cure example demonstrates the application of the methodology to an interval censored outcome on the RMST scale that is easily communicated in a clinical context.

## Supporting information

S1 Appendix Simulation study details. Details of simulation study design.
(PDF)

S1 Table Results for interval censored outcome simulation with narrow intervals. (PDF)
