# Supplementary material for: Causal mediation analysis for time-to-event outcomes on the Restricted Mean Survival Time scale: A pseudo-value approach
Source: PLoS One. 2025 Apr 9;20(4):e0319074. doi: 10.1371/journal.pone.0319074 (PMC11981657; doi:10.1371/journal.pone.0319074)
Supplement: S1 Appendix — Details of simulation study design. (PDF) [file pcbi.0319074.s001.pdf]

S1 Appendix. Causal mediation analysis for time-to-event outcomes on the restricted mean survival time scale: a pseudo-value approach

Ariel Chernofsky[1], Judith J. Lok[2]

1 Department of Biostatistics, Boston University School of Public Health, Boston University, Boston, MA, USA
2 Department of Mathematics and Statistics, Boston University, Boston, MA, USA

* achern@bu.edu

1 Simulation details

The simulation study was designed to emulate the data application of Section 3.2. We also simulated a hypothetical treatment that increases the odds of the viral persistence measure M being below the assay limit by a factor 3.

$$A \sim Bern(0.5),$$

$$C \sim Bern(0.5),$$

$$M \mid A = a, C = c \sim Bern(p_M(a, c)),$$

where

$$\text{logit}(p_M(a, c)) = \text{logit}(P(M = 1 \mid A = a, C = c)) = -0.1 + 1.09a - 0.43c.$$

The survival time, $T \mid A = a, M = m, C = c$ follows a Cox-Weibull model with scale parameter $\lambda = 1.5$ and shape parameter $\nu = 0.8$:

$$h(t \mid A = a, M = m, C = c) = \lambda \nu t^{\nu-1} \exp(-0.2a - m - 0.3c).$$

The RMST for this model is

$$E[\min(T, \tau) \mid A = a, M = m, C = c] = \int_{t=0}^{\tau} \exp\Big(-\lambda t^{\nu} \exp(-0.2a - m - 0.3c)\Big) dt.$$

Thus, we can calculate the necessary elements for the true indirect and direct effects as follows:

$$\begin{aligned} E[\min(T^{(0)}, \tau)] &= E[\min(T, \tau) \mid A = 0] \\ &= \sum_{c=0}^{1} \sum_{m=0}^{1} E[\min(T, \tau) \mid A = 0, M = m, C = c] \\ &\qquad P(M = m \mid A = 0, C = c) P(C = c) \\ &= \sum_{c=0}^{1} \sum_{m=0}^{1} \left(\int_{t=0}^{\tau} \exp\Big(-\lambda t^{\nu} \exp(-m - 0.3c)\Big) dt \right) \\ &\qquad P(M = m \mid A = 0, C = c) P(C = c) \end{aligned}$$

with $P(M=1 \mid A=0, C=c) = \frac{1}{1+\exp(0.1+0.43c)}$,
$P(M=0 \mid A=0, C=c) = \frac{1}{1+\exp(-0.1-0.43c)}$, and $P(C=c) = 1/2$ for $C=0,1$.

$$
\begin{aligned}
E[\min(T^{(1)}, \tau)] &= E[\min(T, \tau) \mid A=1] \\
&= \sum_{c=0}^{1} \sum_{m=0}^{1} E[\min(T, \tau) \mid A=1, M=m, C=c] \\
&\quad P(M=m \mid A=1, C=c) P(C=c) \\
&= \sum_{c=0}^{1} \sum_{m=0}^{1} \left(\int_{t=0}^{\tau} \exp\Big(-\lambda t^{\nu} \exp(-0.2 - m - 0.3c) \Big) dt \right) \\
&\quad P(M=m \mid A=1, C=c) P(C=c)
\end{aligned}
$$

with $P(M=1 \mid A=1, C=c) = \frac{1}{1+\exp(0.1-1.09+0.43c)}$,
$P(M=0 \mid A=1, C=c) = \frac{1}{1+\exp(-0.1+1.09-0.43c)}$, and $P(C=c) = 1/2$ for $C=0,1$.

$$
\begin{aligned}
E[\min(T^{(0,I=1)}, \tau)] &= \sum_{c=0}^{1} \sum_{m=0}^{1} E[\min(T, \tau) \mid M=m, A=0, C=c] \\
&\quad P(M=m \mid A=1, C=c) P(C=c) \\
&= \sum_{c=0}^{1} \sum_{m=0}^{1} \left(\int_{t=0}^{\tau} \exp\Big(-\lambda t^{\nu} \exp(-m - 0.3c) \Big) dt \right) \\
&\quad P(M=m \mid A=1, C=c) P(C=c)
\end{aligned}
$$

with $P(M=1 \mid A=1, C=c) = \frac{1}{1+\exp(0.1-1.09+0.43c)}$,
$P(M=0 \mid A=1, C=c) = \frac{1}{1+\exp(-0.1+1.09-0.43c)}$, and $P(C=c) = 1/2$ for $C=0,1$.
