# Supplementary material for: Causal mediation analysis for time-to-event outcomes on the Restricted Mean Survival Time scale: A pseudo-value approach
Source: PLoS One. 2025 Apr 9;20(4):e0319074. doi: 10.1371/journal.pone.0319074 (PMC11981657; doi:10.1371/journal.pone.0319074)
Supplement: S1 Table — (PDF) [file pcbi.0319074.s002.pdf]

S1 Table. Causal mediation analysis for time-to-event outcomes on the restricted mean survival time scale: a pseudo-value approach

Ariel Chernofsky[1], Judith J. Lok[2]

1 Department of Biostatistics, Boston University School of Public Health, Boston University, Boston, MA, USA
2 Department of Mathematics and Statistics, Boston University, Boston, MA, USA

* achern@bu.edu

1 Simulation results for interval censored outcomes with observed narrow intervals

Interval censored outcome simulation results with narrow intervals. Estimates of the direct, indirect, and total effect of a treatment A on an interval censored outcome T mediated by a binary mediator M on the restricted mean survival time (RMST) scale over an 8-week time horizon. Two estimation procedures were evaluated: 1. Non-parametric Maximum Likelihood Estimate for interval censored data of the RMST 2. Kaplan Meier estimator with event times taken as the midpoints of the intervals. The simulated intervals are based on four scheduled ($K = 20$) visits with two weeks in between visits ($b = 0.5$), random starting times, and a 20% probability of missing each visit. The data were simulated with varying sample sizes (100 or 500). For each sample size, 5000 datasets were simulated. The true direct, indirect, and total effects are 2.8, 3.1, and 5.9, respectively. Scale parameter $\lambda = 1.5$ and shape parameter $\nu = 0.8$.

method[a]	effect	sample size	bias	standard deviation	$\sqrt{\text{MSE}}^b$
NPMLE	direct	100	0.02	3.62	3.62
		500	0.02	1.59	1.59
	indirect	100	-0.01	1.73	1.73
		500	-0.00	0.70	0.70
	total	100	0.01	3.22	3.22
		500	0.02	1.44	1.44
KM midpoint	direct	100	-0.01	3.58	3.58
		500	-0.01	1.58	1.58
	indirect	100	-0.06	1.69	1.69
		500	-0.06	0.68	0.69
	total	100	-0.08	3.17	3.18
		500	-0.07	1.42	1.42

[a] Estimation methods based on pseudo-values:
1. *NPMLE*: Non-parametric Maximum Likelihood Estimate for interval censored data
2. *KM midpoint* Kaplan Meier estimator with event times imputed as the midpoints of the intervals.

[b] Square root of mean squared error.
